# Identification of the key immune and inflammatory related gene CALCRL as diagnostic biomarker in differentiating uterine leiomyosarcoma from leiomyoma

**DOI:** 10.3389/fcell.2026.1777897

**Published:** 2026-04-10

**Authors:** Shiying Chen, Zhuna Wu, Congmei Yang, Li Huang, Yajing Xie, Zhimei Zhou, Liying Sheng, Yueli Wang, Binbin Chen, Shunlan Liu, Yumin Ke

**Affiliations:** 1 Department of Gynecology and Obstetrics, The Second Affiliated Hospital of Fujian Medical University, Quanzhou, Fujian, China; 2 Department of Ultrasound, The Second Affiliated Hospital of Fujian Medical University, Quanzhou, Fujian, China

**Keywords:** CALCRL, immunity, inflammation, uterine leiomyomas (ULM), uterine leiomyosarcoma (ULMS)

## Abstract

**Background:**

Uterine leiomyosarcoma (ULMS) is a highly malignant tumor with a poor prognosis. This study aims to explore the potential and significance of novel immune and inflammation-related diagnostic biomarkers in differentiating ULMS from uterine leiomyomas (ULM).

**Methods:**

We analyzed 25 samples of ULMS and 25 samples of ULM from the GEO database (GSE64763). Differentially expressed genes (DEGs) were identified using R software. Different Inflammation- and immunity-related genes (DIIRGs) were derived by intersecting with immune-related and inflammation-related gene sets. Functional enrichment analysis was conducted on DIIRGs utilizing the Gene Ontology (GO) and Kyoto Encyclopedia of Genes and Genomes (KEGG) databases. The Protein-protein interaction (PPI) networks were employed to investigate the interrelationships among various DIIRGs. Two machine learning algorithms were employed for the selection of diagnostic biomarkers. The diagnostic ability was evaluated through receiver operating characteristic (ROC) curves, principal component analysis (PCA), and a nomogram. To further validate our findings, we assessed the diagnostic value of candidate biomarkers in the validation group, including three datasets (GSE9511, GSE68295, and GSE36610), and performed immunohistochemistry (IHC) in clinical tissue samples. Additionally, this study utilized the Cibersort algorithm to determine the composition patterns of 22 immune cell types within ULMS and analyzed correlations between diagnostic markers and immune cells.

**Results:**

A total of 1,363 DEGs and 12 DIIRGs were identified between ULMS and ULM. GO analysis revealed that DIIRGs were predominantly enriched in the positive regulation of the release of sequestered calcium ions into the cytosol, cytokine activity, and G protein-coupled receptor binding. KEGG analysis indicated enrichment in several signaling pathways, including cytokine-cytokine receptor interaction, chemokine signaling pathway, neuroactive ligand-receptor interaction, and IL-17 signaling pathway. CALCRL was identified as a potential diagnostic biomarker for ULMS based on machine learning algorithms, demonstrating an area under the curve (AUC) of 0.898. Its low expression correlates with ULMS progression, which was corroborated in the validation cohort (AUC = 0.792) and IHC. Immune infiltration analysis revealed that levels of Macrophages M0 and activated mast cells were elevated in ULMS compared to ULM, whereas levels of activated NK cells, resting mast cells, and Neutrophils were significantly reduced. Furthermore, CALCRL expression exhibited a positive correlation with CD4 memory resting T cells and resting mast cells but a negative correlation with CD8 T cells (P < 0.05).

**Conclusion:**

Inflammation and immunity play a pivotal role in the pathogenic mechanism of ULMS. Our study findings suggest that CALCRL can serve as an immune-inflammatory biomarker for ULMS, providing a new perspective for exploring the development and diagnosis of ULMS.

## Introduction

Uterine leiomyosarcoma (ULMS) is the most common pathological type of uterine sarcoma, characterized by a high recurrence rate, strong metastasis, and poor prognosis (Yang et al., 2024a). Despite surgery and standard postoperative adjuvant chemotherapy, the 5-year survival rate for most patients is only 25%–76% (Yang et al., 2024a). Several drugs, such as doxorubicin and tribetidine, have been approved for use in sarcoma patients and have achieved certain clinical efficacy. However, due to the acquisition of chemotherapy resistance, some patients have limited therapeutic effects and benefits. In addition, considering that ULMS and uterine leiomyoma (ULM) can both present with menstrual abnormalities, anemia, pelvic pressure, and urinary or bowel symptoms (Lawlor et al., 2020), and have some common clinicopathological and radiological features (Roberts et al., 2018), there are still some difficulties in differentiating these two diseases, especially in preoperative diagnosis at present. Due to the difference in treatment strategies between the two diseases, an incorrect evaluation can lead to delayed treatment or inappropriate treatment. Therefore, it is important to explore and identify sensitive, efficient, and specific diagnostic biomarkers associated with ULMS.

Nowadays, there has been an increasing number of studies exploring the relationship between immunity and inflammation in tumor development. The interactions between tumor cells, immune cells, and cytokines in the tumor immune microenvironment (TIME) determine the direction of tumor development. Immune evasion, one of the main characteristics of tumors, can promote the rapid growth and metastasis of tumor cells. Immune checkpoint inhibitors (ICIs) and adoptive cell transfer (ACT) have shown promising application prospects (Rui et al., 2023). Inflammatory cells and inflammatory mediators have also been shown to be an important component of the local tumor environment. Chronic inflammation can induce gene mutations, promote angiogenesis and cell proliferation, and change the expression of proto-oncogenes and tumor suppressor genes to exert its carcinogenic effects (Kim and Seki, 2025; Soler et al., 2023). Meanwhile, inflammation and immunity also interact with each other. Under different conditions, inflammation can promote immune responses, kill pathogens, promote tissue repair, and inhibit tumor growth. It can also recruit various immunosuppressive cells, such as MDSC and Treg, to promote the establishment of an immunosuppressive tumor microenvironment (TME), leading to immune suppression and thus promoting tumor development (Zhao et al., 2021). At the same time, immune cells can regulate the expression of various inflammatory cytokines and cytokines such as IL-6, THF-α, IL-17, and IL-22, to regulate inflammation (Lv et al., 2022), thereby affecting the growth of tumor cells.

Currently, a variety of novel immunotherapies and inflammation regulation techniques are being discovered, validated, and gradually applied in clinical practice, especially for some difficult-to-treat cancer patients, achieving certain therapeutic effects and improving the prognosis of these groups to some extent. Considering the poor prognosis of ULMS, we hope that through this study, we can find immune and inflammation diagnostic markers and possible target sites related to ULMS, in order to improve the accuracy of ULMS diagnosis and provide a certain direction and basis for exploring new treatment methods for it.

## Methods

### Data collection and processing

We obtained the Training group dataset GSE64763 (including 25 ULM and 25 ULMS samples) from the GEO database. To independently validate our findings, we constructed a validation cohort by merging three separate datasets: GSE9511, GSE68295, and GSE36610. This combined validation cohort contained a total of 10 ULM and 24 ULMS samples (Table 1). We note that while the dataset GSE36610 contributed 12 ULMS samples but no ULM samples, it is profiled on a distinct microarray platform (GPL7363). Its intentional inclusion allows for a stringent test of the cross-platform consistency and robustness of candidate biomarkers, moving beyond validation within a single technical framework. The three validation datasets were merged into a single metadata cohort, and potential batch effects were removed using the “SVA” package in R software to ensure comparability.

**TABLE 1:** mRNA expression profiles related to ULMS and ULM.

Dataset ID	Platform	ULMS	ULM
Training group			
GSE64763	GPL571-17391	25	25
Validation group			
GSE9511	GPL80-30376	9	7
GSE68295	GPL6480-9577	3	3
GSE36610	GPL7363-11635	12	0

### Identification and functional enrichment of DIIRGs

The identification of DEGs was conducted using the “Limma” software package in the R package. DEGs were selected based on |log fold change (Fc)| = 0.585 and adj.P.Val.Filter < 0.05. A heatmap was drawn to display the DEGs, and a volcano plot was drawn using the R package ggplot2. To determine the DIIRGs in ULMS, we conducted an intersection between immune-related genes, inflammation-related genes, and DEGs. We obtained immune-related gene data from the ImmPort database (https://www.immport.org/shared/) (Supplementary Table S1) and identified 200 inflammatory response-related genes from the Molecular Signatures database (https://www.gsea-msigdb.org/gsea/msigdb) (Supplementary Table S2). Subsequently, an intersection was carried out among the IRGs, genes related to inflammation, and DEGs, thus enabling the identification of DIIRGs in ULMS. Using the R packages “enrichplot,” “org.Hs.e.g.,.db,” “clusterProfiler,” and “DOSE” to conduct GO and KEGG pathway enrichment analyses on the DIIRGs. The goal was to identify the GO terms enriched in three categories: biological processes (BP), cellular components (CC), and molecular functions, along with the KEGG pathways. To visualize these enrichment results, the “ggplot2” package in R was utilized. GO and KEGG enrichment analyses were performed on DEGs with a significance threshold of P < 0.05.

### PPI network construction and analysis

The STRING website (https://string-db.org/) was utilized to search for a PPI network using 12 DIIRGs in the “multiple proteins” module and “*Homo sapiens*” in the organism module. We extracted gene symbols from protein IDs and filtered out PPIs lacking corresponding gene names. Subsequently, we used Cytoscape 3.10.0 to construct the PPI network. Moreover, the cytoHubba plugin helped us identify the hub genes. Finally, key genes that play a role in the development of ULMS were selected.

### Screening biomarkers for ULMS by machine learning algorithms

We pinpointed the significant diagnostic biomarkers in ULMS by using Spearman correlation analysis to select DIIRGs with a |log fold change (FC)| value of 0.585. The discovery of these biomarkers was facilitated by the use of the Least Absolute Shrinkage and Selection Operator (LASSO) algorithm in combination with the multiple support vector machine recursive feature elimination (mSVM-RFE). LASSO was used for feature selection to prevent model overfitting. alpha = 1 specifies LASSO regression. Through 10-fold cross-validation, the model’s performance under different λ values was evaluated on the training data. The optimal λ value (lambda.min) that minimizes the model’s deviance was ultimately selected to identify the feature genes most contributive to ULMS classification. Additionally, we implemented the mSVM-RFE algorithm using the “e1071” R package. The mSVM-RFE algorithm was used for stable feature selection. This method evaluates feature importance through recursive feature elimination combined with cross-validation. In the script, 10-fold cross-validation was applied to assess feature subsets and select the optimal gene set for distinguishing ULMS from ULM. The algorithm aims to reduce overfitting by leveraging resampling during feature selection. Since mSVM-RFE has a reduced risk of overfitting in comparison to SVM-RFE, we combined LASSO and mSVM-RFE to screen for overlapping genes. Subsequently, we validated these genes within the validation dataset (GSE9511 + GSE68295 + GSE36610). Finally, we used the R package “pROC” to perform ROC analysis on the dataset and calculate the AUC value to determine whether the expected biomarker can effectively distinguish ULMS from ULM.

### Construct and validate the nomogram model for assessing the diagnostic efficacy of ULMS

A nomogram model was constructed using the “rms” and “rmda” packages to predict the occurrence of ULMS. Each factor’s score is denoted as “points,” while the cumulative score of all factors is referred to as the “total points.” Subsequently, calibration curves were generated to evaluate the predictive performance of the nomogram model.

### Evaluating the level of immune infiltration in ULMS and biomarkers

The CIBERSORT algorithm (http://cibersort.stanford.edu/) was used to quantify the relative abundances of 22 immune cell types in ULMS and ULM samples based on gene expression data. Support vector regression was used for the deconvolution calculation. The statistical significance (P-value) of the immune infiltration results for each sample was assessed through 1,000 permutation tests to ensure the reliability of the findings. The Vioplot package was used to study the penetration differences between ULMS and ULM groups. Pearson’s correlation analysis was utilized to investigate the association between screened diagnostic biomarkers and the levels of immune-infiltrating cells, and the outcomes were visualized using the “ggplot2” R package.

### Patient and tissue samples

From May 2016 to December 2023, at The Second Affiliated Hospital of Fujian Medical University (Fujian, China), gynecological oncology pathologists diagnosed twenty-one paraffin-embedded ULMS and thirty-eight ULM specimens. All patients mainly received hysterectomy with/or bilateral adnexal resection as treatment. Before the study commenced, the Research Ethics Committee of The Second Affiliated Hospital of Fujian Medical University approved the research.

### Immunohistochemistry (IHC)

We used the primary antibody is anti-CALCRL (ZEN-BIOSCIENCE, Chengdu). We classified the staining intensity ratios of CALCRL into four levels: negative (scored as 0 points), light yellow (1 point), brownish yellow (2 points), and tan (3 points). For the number of stained cells, we evaluated it according to the following proportions: if less than one-third, we assigned one point; if between one-third and two-thirds, two points; and if more than two-thirds, three points. We calculated the final expression scores of CALCRL by multiplying the two aforementioned ratings. Subsequently, we divided the slide samples into two groups, namely, the low-expression group and the high-expression group. The low-expression group was defined by scores of less than 6, and the high-expression group by scores of greater than or equal to 6. Two pathologists specialized in gynecological oncology validated the histopathological diagnoses of the patients.

### Statistical methods

All statistical analyses were performed using R software (v.4.1.1). We compared the ULMS group with the ULM group using the Mann–Whitney U test. The analyses involved LASSO regression, the SVM-RFE algorithm, ROC analysis, Pearson’s correlation, and unpaired t-tests. Statistical significance was set at P < 0.05, and significance levels were denoted as *P < 0.05, **P < 0.01, and ***P < 0.001. Detailed parameters for all bioinformatics analyses, including differential expression analysis, LASSO regression, SVM-RFE algorithm, and CIBERSORT immune infiltration, are provided in Supplementary Table S3.

## Results

### Study procedure

**FIGURE 1:**
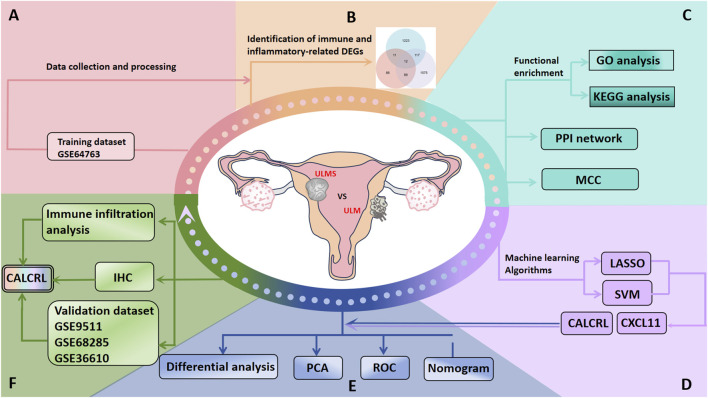
The flowchart for the study’s overall workflow.

Figure 1 shows the flowchart of the overall study. We obtained transcriptome RNA-seq data from the GEO database, and DEGs were identified through differential gene expression analysis of the training datasets. Subsequently, we conducted an intersection analysis with immune gene and inflammation gene sets to obtain DIIRGs. Enrichment analyses were conducted using GO and KEGG. Additionally, candidate overlapping genes were further refined via PPI networks alongside two machine learning algorithms: LASSO and SVM-RFE. The predictive capacity of the biomarkers was assessed using PCA, ROC curves, and a nomogram, which were subsequently validated in an independent dataset comprising GSE9511, GSE68295, and GSE36610. We also evaluated the composition patterns of 22 immune cell types in ULMS utilizing the Cibersort algorithm, followed by a correlation analysis between the selected diagnostic markers and immune cells. Finally, IHC staining was performed on paraffin-embedded specimens that met predefined inclusion criteria to validate our findings.

### Identification of DIIRGs in ULMS

**FIGURE 2:**
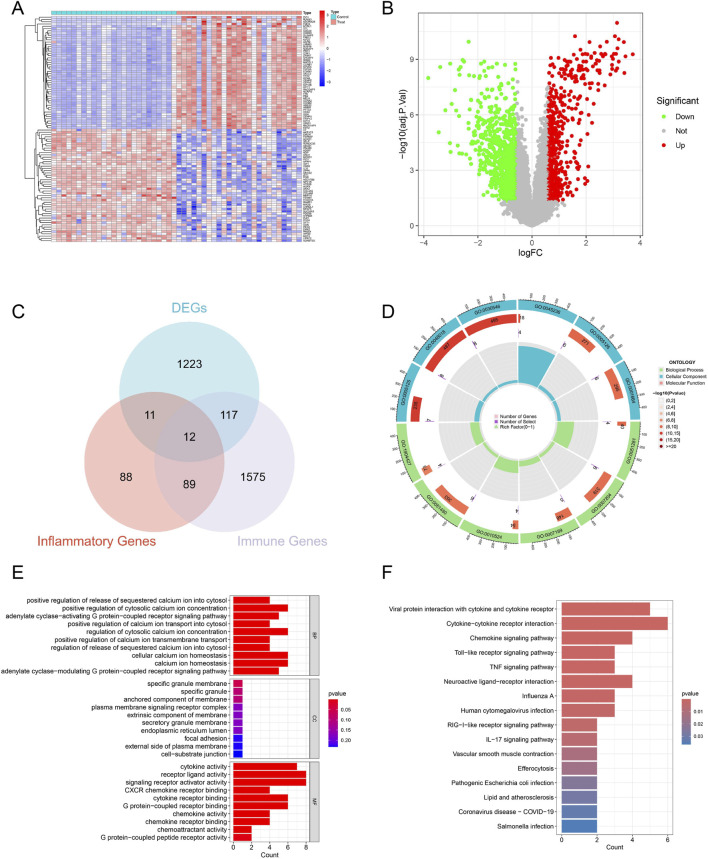
Detection and function enrichment analysis of DIIRGs between ULMS and ULM. **(A)** The heatmap presents the significant DEGs. **(B)** Volcano plot shows the distribution of DEGs. Red represented a high expression of robust DEGs, while green represented a low expression of robust DEGs. **(C)** A Venn plot was used to identify the common immune and inflammatory-related DEGs. **(D)** A circle plot for GO analysis of 12 DIIRGs is presented. Biological processes (BP), cellular components (CC), and molecular functions (MF). **(E)** A bar graph for GO analysis of 12 DIIRGs is shown. **(F)** The 12 DIIRGs are annotated by KEGG. The y-axis is the KEGG pathway enriched terms, and the x-axis is the fold of enrichment.

Our study identified 1,363 DEGs by comparing 25 ULM samples with 25 ULMS samples from the GSE64763 dataset. Among these genes, 774 showed significant downregulation and 589 had significant upregulation (Figures 2A,B) (Supplementary Table S4). As shown in Figure 2C, we identified 12 DIIRGs by intersecting the DEGs, IRGs, and inflammatory genes (Supplementary Table S5). In the ULMS group, the expression of ADM, PLAUR, LIF, CXCL8, CXCL10, and CXCL11 was elevated, whereas the expression of CALCRL, NDP, APLNR, TNFSF10, PTGER2, and CX3CL1 exhibited marked downregulation.

### Functional enrichment analysis

Enrichment analysis was employed to investigate the biological functions of the 12 DIIRGs. The GO analysis revealed that these DIIRGs were predominantly enriched in positive regulation of release of sequestered calcium ion into cytosol, cytokine activity, G protein-coupled receptor binding, and tumor necrosis factor receptor superfamily binding (Figures 2D,E; Supplementary Table S6). Furthermore, KEGG analysis indicated enrichment in several signaling pathways, including cytokine-cytokine receptor interaction, chemokine signaling pathway, neuroactive ligand-receptor interaction, Toll-like receptor signaling pathway, TNF signaling pathway, and IL-17 signaling pathway (Figure 2F; Supplementary Table S7). These findings will facilitate further exploration of the potential mechanisms underlying ULMS development.

### PPI network construction and hub gene selection

**FIGURE 3:**
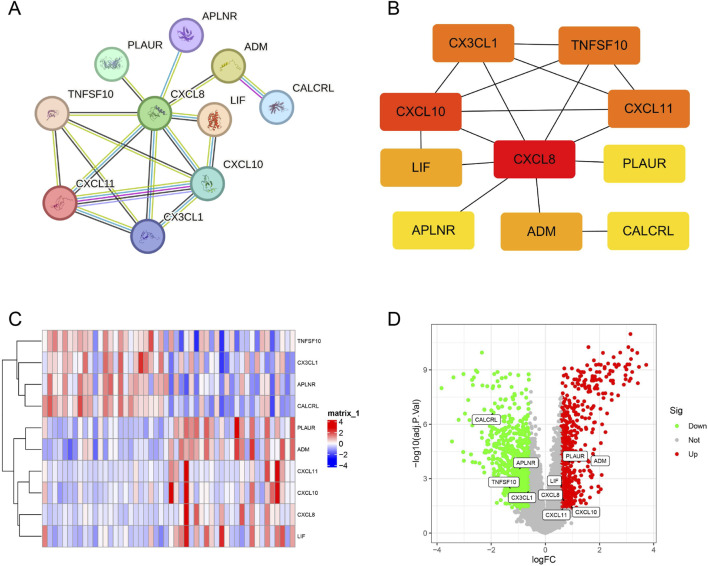
Correlation of the core DIIRGs. **(A)** The PPI network was constructed by the 10 common DIIRGs. **(B)** A network diagram depicting 10 core DIIRGs was acquired via the MCC algorithm. **(C)** Heatmap and **(D)** volcano plot presenting the 10 hub DIIRGs.

The interactions among 12 DIIRGs were explored by leveraging the STRING database (https://string-db.org/). By doing so, PPI networks with hidden isolated nodes removed were constructed. Subsequently, it was found that ten genes, namely, ADM, PLAUR, LIF, CXCL8, CXCL10, CXCL11, CALCRL, APLNR, TNFSF10, and CX3CL1, were interconnected (Figure 3A). Next, the cytoHubba plugin within the Cytoscape software was employed to carry out gene clustering in the network. As a result, ten nodes related to the MCC were detected and grouped (Figure 3B). Significantly, four of these genes (CALCRL, APLNR, TNFSF10, and CX3CL1) exhibited reduced expression levels, whereas six of these genes (ADM, PLAUR, LIF, CXCL8, CXCL10, and CXCL11) demonstrated an upregulated expression in the ULMS group (Figures 3C,D).

### Correlation of prospective biomarkers

**FIGURE 4:**
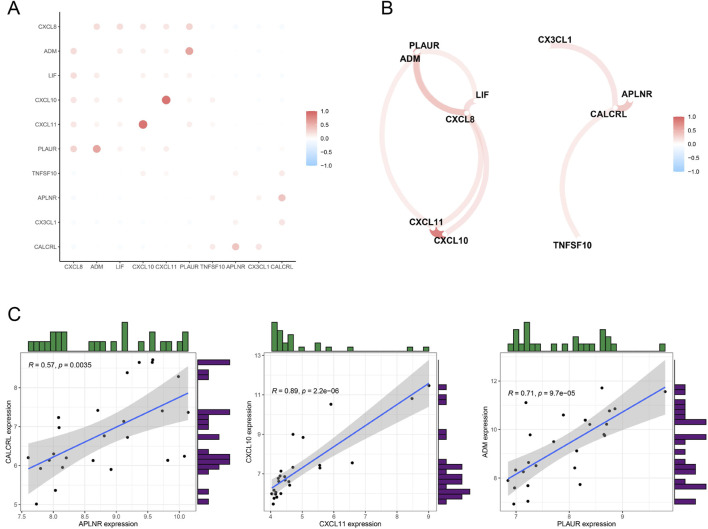
Exploring Correlations Among DIIRGs in ULMS. **(A)** A co-expression network map of DIIRGs is presented. **(B)** The correlations among DIIRGs are visualized in a plot. **(C)** A scatter plot is provided to illustrate some DIIRGs with high correlation.

We conducted a correlation analysis on the DIIRGs to explore the associations between their expression levels using a cutoff of 0.3 and pFilter = 0.05. To visually represent our findings, we employed the “tidyverse” and “corrr” packages in the R programming language, which facilitated the generation of a co-expression correlation heatmap (Figure 4A) and a correlation network diagram (Figure 4B). Furthermore, we also created scatter plots for the 10 gene groups exhibiting the strongest correlations (with a correlation coefficient cutoff of 0.5 and a P value less than 0.05) in ULMS patients (Figure 4C). The analysis revealed that CALCRL has a positive association with both APLNR (R = 0.57). Similarly, a positive correlation was detected between CXCL10 and CXCL11 (R = 0.89), and PLAUR is positively correlated with ADM (R = 0.71).

### A prediction model for ULMS is built

**FIGURE 5:**
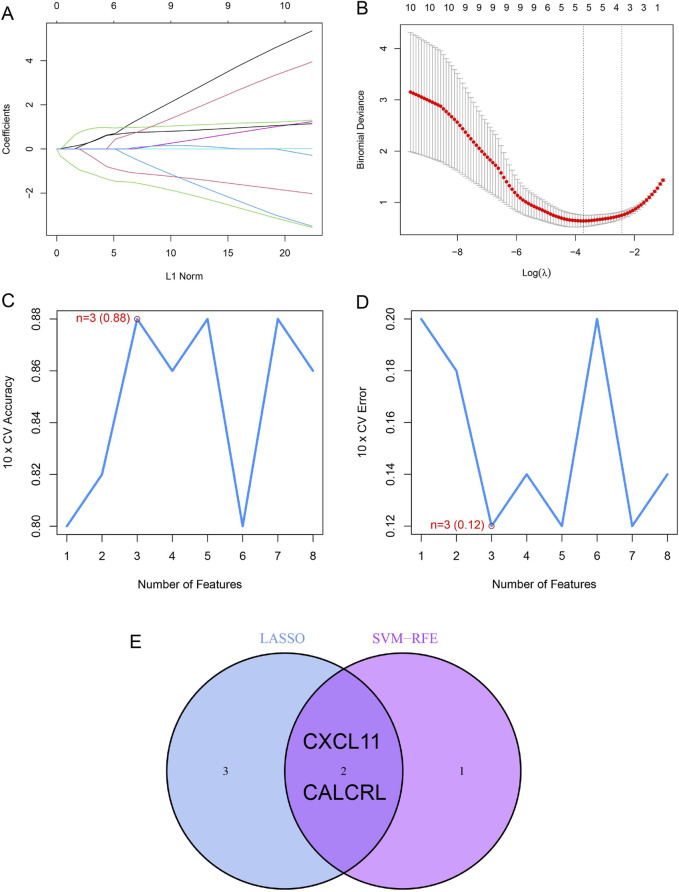
Further Screening process of DIIRGs for ULMS diagnosis. **(A)** A curve illustrating the LASSO regression coefficient profiles of the 5 DIIRGs depicts the variation trajectory of each DIIRG. **(B)** The LASSO Cox regression model was used to generate a plot of partial likelihood deviance against log(λ). **(C)** When k = 3, the curve of total within-cluster sum of squared errors (WSS) corresponding to the cluster number k reaches the “elbow point.” **(D)** At k = 3, the curve representing the average silhouette width for the corresponding cluster number k attains its maximum value. **(E)** A Venn diagram exhibits two diagnostic markers that are common to both the LASSO and SVM-RFE algorithms.

In order to precisely determine the crucial prognostic biomarkers for ULMS, the LASSO and mSVM-RFE algorithms were employed. As demonstrated in Figures 5A,B, via LASSO analysis, five DIIRGs—CXCL11, APLNR, PLAUR, ADM, and CALCRL—were selected from a set of ten DIIRGs associated with ULMS. Concurrently, by applying the mSVM-RFE model, the set of ten DIIRGs was reduced to three DIIRGs, namely, CALCRL, CXCL11, and CXCL8, as shown in Figures 5C,D. Upon comparing the outcomes of the two algorithms, it was observed that the final selection comprised three overlapping candidate genes: CXCL11 and CALCRL, as presented in Figure 5E.

### Effectiveness of diagnostic biomarkers in ULMS

**FIGURE 6:**
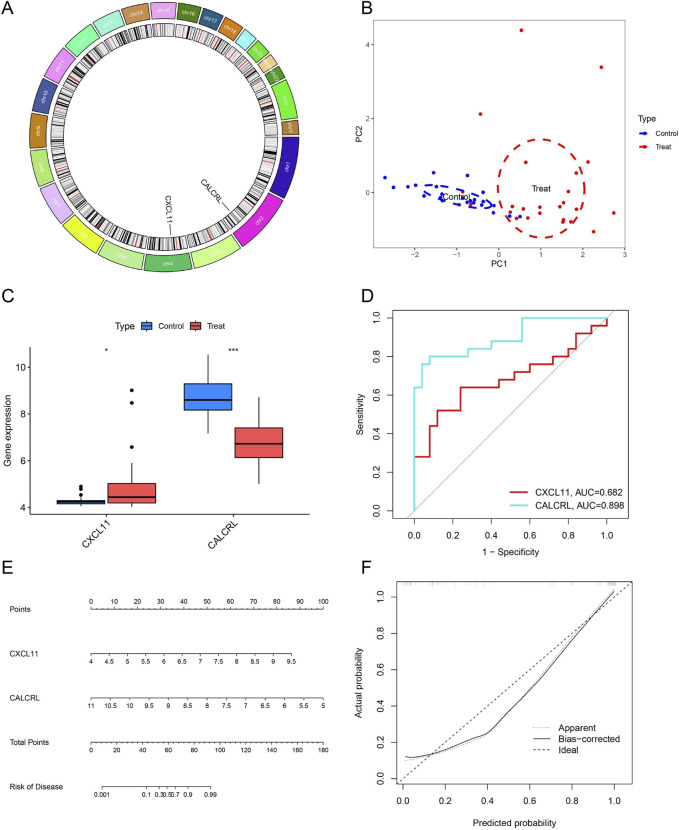
Supplementary Analysis of Two Key DIIRGs. **(A)** Chromosomal locations of the two key DIIRGs are presented. **(B)** PCA based on the three aforementioned genes enables clear differentiation between ULMS and ULM samples. **(C)** The GSE64763 dataset illustrate the relative expression levels of thetow key DIIRGs in ULMS versusULM samples. **(D)** ROC curves were utilized to verify the performance of the two key DIIRGs in predicting ULMS within the GSE64763 dataset. **(E)** A diagnostic nomogram associated with the two key DIIRGs is presented. **(F)** A calibration curve is shown for the prediction model composed of the two key DIIRGs.

Figure 6A displays the genomic positions of CXCL11 and CALCRL on chromosomes. As illustrated in Figure 6B, the results of the principal component analysis suggest that the two candidate genes are highly proficient in differentiating between ULMS and ULM, indicating their potentially vital role in the diagnosis of ULMS. Furthermore, CALCRL was found to have decreased expression levels, and CXCL11 has increased expression levels in ULMS (Figure 6C). ROC analysis was performed on potential DIIRGs to assess their predictive accuracy. The results indicated that the AUC value for the CALCRL gene was 0.898, while the AUC value for the CXCL11 gene was 0.682, suggesting that the CALCRL gene has a superior predictive power for ULMS compared to ULM, as seen in Figure 6D. A nomogram was used to assess the diagnostic efficacy and predictive capability of the key DIIRGs for ULMS. Subsequently, a nomogram for ULMS, which was linked to risk, was developed (Figures 6E,F). It could act as an approach to evaluate the efficacy of the risk score in distinguishing ULMS from ULM.

### Verification of the crucial DIIRGs

**FIGURE 7:**
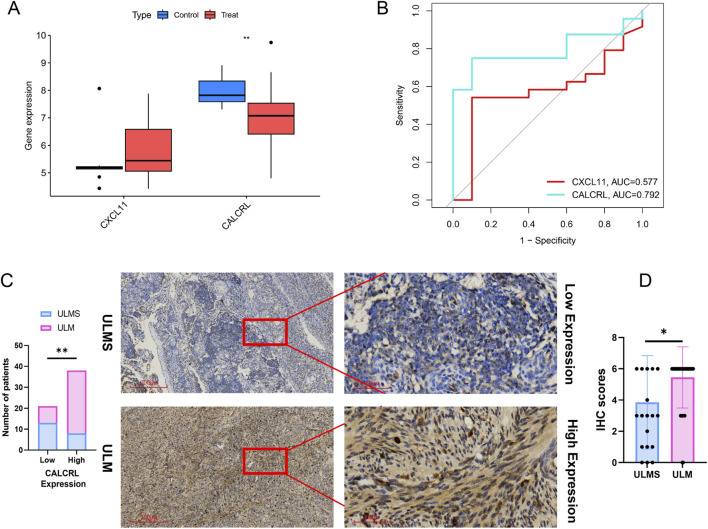
Validation of the Two Essential DIIRGs. **(A)** A boxplot illustrates the expression levels of DIIRGs between ULMS and ULM in the test cohort (GSE9511+GSE68295+GSE36610); **P < 0.01. **(B)** A ROC curve was generated to verify the diagnostic efficacy of the two DIIRGs in the test group. Compared with ULM specimens (n = 5), LYN exhibited significantly lower expression in RSA tissues (n = 6). **(C)** Stratification of 21 ULMS patients and 38 ULM patients based on CALCRL expression levels is presented. A bar plot shows the count of ULMS and ULM patients categorized by CALCRL expression (Low vs. High), with the y-axis representing the number of patients. Chi-square test was used to compare the distribution differences between ULMS and ULM in each CALCRL expression group, with statistical significance set at P < 0.05 (denotes **P < 0.01.). Representative IHC staining images of CALCRL in ULMS and ULM specimens (magnification: ×40 and ×200) are also shown, displaying high and low expression levels. **(D)** Semi-quantitative analysis of CALCRL immunohistochemistry (IHC) scores in ULMS (n = 21) and ULM (n = 38) clinical tissue samples. The IHC score was calculated by multiplying staining intensity (0–3) by the proportion of positive cells (1–3), resulting in a total score ranging from 0 to 9. Each dot represents an individual sample; horizontal lines indicate the mean ± SD. Statistical significance was determined using the Mann–Whitney U test. *P < 0.05 indicates a significantly lower CALCRL expression in ULMS compared to ULM.

Furthermore, to ensure a more reliable and accurate selection of DIIRGs, we validated the expression levels of these candidates using an independent validation dataset. The results indicated that CALCRL expression was significantly reduced in ULMS tissue compared to ULM tissue, while no significant difference was noted for CXCL11 between the two groups (Figure 7A). The AUC values of the ROC curves for two DIIRGs (AUCCXCL11 = 0.577; AUCCALCRL = 0.792) demonstrated that CALCRL has a superior predictive power for ULMS compared to ULM (Figure 7B). These findings reinforce the conclusion that CALCRL may serve as a promising diagnostic biomarker for ULMS. To conduct further clinical validation, IHC was employed to measure the expression of CALCRL. The findings revealed that ULMS was associated with low expression levels of CALCRL (Figure 7C; P < 0.05). IHC scores comparing CALCRL expression between ULMS (n = 21) and ULM (n = 38) samples. As shown in the Figure 7D, the IHC scores (mean ± SD) for ULMS (3.857 ± 2.988) were significantly lower than those for ULM (5.447 ± 1.955), with P = 0.0157. Overall, these results suggest that the CALCRL gene has greater diagnostic value in ULMS.

### Evaluating the level of immune cell infiltration in ULMS

**FIGURE 8:**
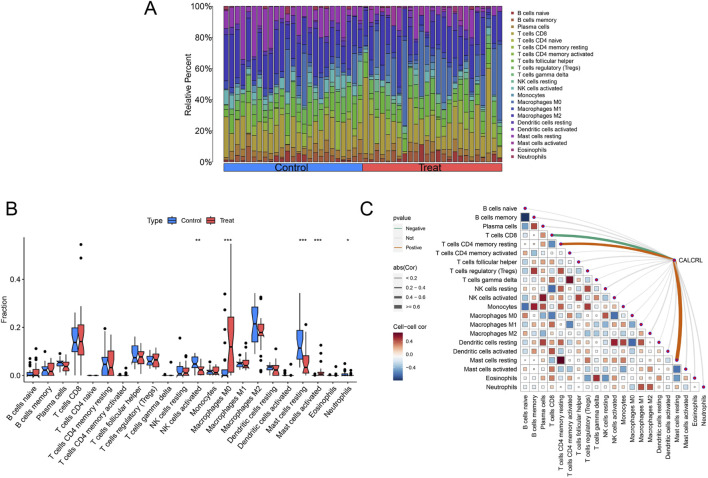
The evaluation of ULMS-related immune infiltration. **(A)** A barplot illustrates the expression levels of DIIRGs between ULMS and ULM in the test cohort (GSE64763). **(B)** Differential immune cell infiltration profiles between ULMS and ULM. **(C)** DIIRGs-immune cell correlation network diagram: It shows the interactions between the CALCRL gene and 22 types of immune cells in ULMS. All significant lines have passed statistical tests (P < 0.05). *P < 0.05, *P < 0.01, ***P < 0.001.

We employed the CiberSort algorithm to quantify the proportions of 22 immune cell types in ULMS and ULM samples (Figure 8A). Subsequently, we analyzed the differences in immune cell composition between the two groups. The results indicated that the proportion of M0 macrophages and activated mast cells was significantly higher in the ULMS group compared to the ULM group, whereas activated NK cells, resting mast cells, and neutrophils were significantly less prevalent in the ULMS group (Figure 8B). Furthermore, we investigated the correlation between diagnostic biomarkers associated with various infiltrating immune cells. The findings revealed that CALCRL expression exhibited a positive correlation with CD4 memory resting T cells and resting mast cells, while showing a negative correlation with CD8 T cells (P < 0.05) (Figure 8C). These results underscore that CALCRL is closely linked to immune activity and has significant implications for regulating immune cell dynamics in ULMS.

## Discussion

ULMS constitutes approximately 5% of all uterine malignant tumors, with smooth muscle leiomyosarcoma being the most prevalent subtype, accounting for about 30%. Due to its propensity for metastasis and the development of chemotherapy resistance, this tumor is associated with a high mortality rate (Yang et al., 2024b). Although there have been increasing numbers of studies exploring the molecular environment of smooth muscle leiomyosarcoma and identifying chromosomal aberrations or heterozygous deletions (chromosomes 2p, 2q, 10q, 13q) and dysregulated genes (TP53, RB1, PTEN, MED12, YWHAE, VIPR2) associated with the disease, the pathogenesis and molecular events of most leiomyosarcomas remain unknown (Sanegre et al., 2021).

With the ongoing advancement of bioinformatics technologies, a substantial amount of sample data derived from databases such as GEO and TCGA has been generated, leading to the identification of numerous immune and inflammation-related diagnostic and therapeutic targets for tumors that are anticipated to be translated into clinical applications. The progression of ULMS is also intricately linked to the regulation of inflammation and immunity. Tsai-Der Chuang, PhD, and colleagues investigated the role of miR-200c in regulating inflammation-related genes in ULMS. They found that miR-200c expression was significantly lower in leiomyosarcomas than in leiomyomas, leading to reduced control over target genes (e.g., IL-8, IKBKB, CDK2, CCNE2) and altered NF-kB signaling pathway activity (Chuang et al., 2015). Consequently, this dysregulation promotes pro-inflammatory responses, angiogenesis, and cell cycle progression—ultimately facilitating sarcomatous transformation from leiomyoma to leiomyosarcoma (Chuang et al., 2015). We identified 12 overlapping DIIRGs by cross-analysis of DEGs with IRGs and inflammatory genes. KEGG analysis of these DIIRGs revealed significant enrichment in the TNF signaling pathway as well as other inflammation-related pathways, including IL-17. Pelly et al. demonstrated that in CT26 colon cancer cells and low-immunogenic 4T1 breast cancer cells, the application of cyclooxygenase-2 (COX-2) inhibitors or prostaglandin E2 (PGE2) receptor antagonists could activate IFN-γ-driven transcriptional reprogramming, thereby enhancing the accumulation of effector T cells within tumors. This resulted in heightened responsiveness to immunotherapy, ultimately leading to tumor progression suppression and substantial survival benefits (Pelly et al., 2021). These findings suggest that synergistic immunotherapy targeting anti-inflammatory pathways may offer new hope for ULMS patients as well as those with tumors resistant to conventional immunotherapies.

Immune infiltration has been confirmed to occur in the majority of ULMS cases. The immune microenvironment of ULMS is characterized by a substantial presence of tumor-associated macrophages (TAM) and tumor-infiltrating lymphocytes (TIL). The abundance and density of TAMs are associated with poor prognosis in ULMS, whereas higher TIL density correlates with improved progression-free survival (PFS) and overall survival (OS) (Cope et al., 2023). Additionally, regulatory T cells (Tregs), myeloid-derived suppressor cells (MDSCs), and other immunosuppressive cell types are present within the ULMS microenvironment, contributing to anti-tumor immune responses (De Wispelaere et al., 2021). Checkpoint inhibition therapy (ICB) targeting the PD1/PDL1 pathway has demonstrated efficacy in treating ULMS, albeit with limited effectiveness. Notably, the positive expression rate and levels of PDL-1 are significantly elevated in high-grade advanced ULMS, suggesting a tumor microenvironment characterized by heightened inflammation and immune infiltration (Cope et al., 2023). Research conducted by Shen et al. indicates that infiltration levels of memory B cells, M0 macrophages, activated mast cells, and follicular helper T cells in ULMS are markedly higher than those observed in normal smooth muscle cells (Shen et al., 2021). Our study also identified significant infiltration of M0 macrophages and activated mast cells within ULMS. Characterizing the cellular composition involved in immune suppression within the ULMS tumor microenvironment will provide valuable insights for exploring novel immune-assisted therapeutic strategies.

We employed two machine learning algorithms to integrate and validate two candidate genes (CALCRL and CXCL11), utilizing a validation dataset (GSE9511 + GSE68295 + GSE36610) to confirm our findings, ultimately establishing CALCRL as a potential diagnostic biomarker for ULMS. CALCRL is extensively distributed throughout the human body, particularly in endothelial cells of capillaries, arterial smooth muscle cells, and immune cells (Wende et al., 2023). In addition to its role in vascular regulation, CALCRL functions as both a mineralocorticoid receptor and a CGRP receptor, mediating various biological activities. Currently, CALCRL is recognized as being involved in the pathogenesis of various diseases, including thyroid cancer, small cell lung cancer, renal clear cell carcinoma, lymphoma, and melanoma (Wende et al., 2023). The CGRP signaling mediated by CALCRL can enhance chemoresistance and stem cell characteristics in acute myeloid leukemia (AML), with CGRP serving to protect AML cell lines and primary AML samples from apoptosis induced by cytotoxic agents used in treatment. The development of novel targeted therapies may offer new hope for patients with refractory leukemia (Gluexam et al., 2019; Grandits and Wieser, 2021). Xiaoyun Bin et al. employed bioinformatics techniques to analyze the expression levels of CALCRL in high-risk liver cancer populations (HR = 0.78) and found that when combined with DEPDC1, DEPDC1B, NGFR, PRR11, and TRIP13, it provided significant predictive value for liver cancer risk stratification (Bin et al., 2023). He and Ling assessed CALCRL expression levels in non-small cell lung cancer (NSCLC) using immunohistochemistry (IHC) and Western blotting (WB), revealing a significant upregulation of CALCRL associated with tumor invasion; knocking down CALCRL markedly inhibited cellular proliferation and migration while promoting apoptosis (He and Ling, 2021). Xing et al. reported decreased expression of CALCRL in cervical adenocarcinoma patients; notably, those exhibiting low expression demonstrated improved overall survival (OS) outcomes (Xing et al., 2022). A systematic comparison of CALCRL expression across different tumor types reveals striking heterogeneity that underscores its context-dependent biological roles (Table 2). Notably, CALCRL exhibits diametrically opposite expression patterns in different malignancies: it is significantly downregulated in ULMS and GBM, but markedly upregulated in NSCLC and AML. This dichotomy suggests that CALCRL may function as a tumor suppressor in some contexts (e.g., ULMS, GBM) while acting as an oncogene in others (e.g., NSCLC, AML). A very recent study further underscores the clinical relevance of CALCRL in AML, demonstrating that its high expression is associated with poor prognosis and may serve as a prognostic biomarker (Khorshied et al., 2026). The specificity of our findings in ULMS is further highlighted by several unique aspects. First, unlike most studies that compare tumor versus normal tissues, we directly compared ULMS with its benign counterpart (ULM), establishing CALCRL as a potential diagnostic biomarker for distinguishing these clinically similar entities. Second, the immune correlation pattern observed in ULMS—positive correlation with CD4^+^ memory resting T cells and resting mast cells, negative correlation with CD8^+^ T cells—appears distinct from other cancers where CALCRL has been primarily studied for cell-autonomous functions such as proliferation, chemoresistance, or stemness. Third, the tissue-specific co-expression of CALCRL with RAMPs in the uterus may confer its downregulation unique pathophysiological consequences related to hypoxia adaptation, lymphatic regulation, and local immune modulation that are not applicable to other organ systems. Collectively, these findings position CALCRL as a context-dependent biomarker whose low expression in ULMS carries diagnostic, immune-modulatory, and potentially therapeutic implications that are distinct from its roles in other malignancies. Given the diverse findings regarding CALCRL across different diseases, we hypothesize that CALCRL may play different roles in different diseases; however, there are currently no studies investigating the relationship between CALCRL and UMLS.

**TABLE 2:** Systematic comparison of CALCRL expression and function across tumor types.

Tumor type	Expression pattern in tumor vs. normal/benign	Functional role/association	Prognostic/diagnostic significance	Key references
Uterine leiomyosarcoma (ULMS)	Significantly downregulated (vs. benign leiomyoma)	Positively correlated with CD4^+^ memory resting T cells and resting mast cells; negatively correlated with CD8^+^ T cells	Diagnostic biomarker for distinguishing ULMS from ULM (AUC = 0.898)	Present study
Glioblastoma (GBM)	Significantly downregulated	CT-CALCRL signaling axis acts as tumor suppression pathway	Low expression correlates with poor prognosis	Pal et al., (2018); Wang et al., (2020)
Non-Small Cell Lung Cancer (NSCLC)	Significantly upregulated	Promotes proliferation and migration; inhibits apoptosis	High expression associated with tumor invasion and poor outcomes	He and Ling, (2021)
Acute Myeloid Leukemia (AML)	Upregulated	Enhances chemoresistance and stem cell properties	High expression linked to therapy resistance, relapse, and poor prognosis; recent study confirms its clinical relevance as a prognostic biomarker	Gluexam et al., (2019), Grandits and Wieser, (2021), Tang et al., (2023), Khorshied et al., (2026)
Cervical Adenocarcinoma	Downregulated	Not fully characterized	Low expression favorable for OS	Xing et al., (2022)
Hepatocellular Carcinoma (HCC)	Variable (context-dependent)	Part of five-gene immune signature for risk stratification	HR = 0.78 (protective factor)	Bin et al., (2023)
Thyroid Cancer/Renal Cell Carcinoma/Melanoma	Expressed	Involved in pathogenesis; precise mechanisms unclear	Not well established	Wende et al., (2023)

As an immune-related gene, CALCRL plays a crucial role in regulating macrophage function by mediating the CGRP signaling pathway, which is involved in the regulation of cytokine and chemokine production (Ma et al., 2010). Its mediated adrenomedullin signaling pathway is thought to be involved in lymphangiogenesis, thereby nourishing fluid homeostasis, immune defense, and tumor metastasis (Fritz-Six et al., 2008). Consistent with its vascular and lymphatic regulatory roles, recent evidence shows that adrenomedullin overexpression confers protection against pulmonary hypertension and lung injury (Thapa et al., 2025), further supporting the therapeutic potential of targeting this pathway. Research conducted by JING-JING Wang et al. demonstrates that CALCRL expression is significantly downregulated in glioblastoma (GBM) (HR: 0.943), positioning it as one of the survival-related immune-related genes (IRGs) within this disease and contributing to the establishment of GBM risk and prognosis models (Wang et al., 2020). Mutations or reduced transcription levels of CALCRL are often associated with poor prognostic outcomes of GBM. The exogenous administration of calcitonin (CT) suppresses various characteristics of glioma cells and oncogenic signaling pathways in a CALCRL-dependent manner, thus inhibiting tumor growth. These findings underscore the CT-CALCRL signaling axis as a significant tumor suppression pathway and suggest its potential as a novel therapeutic target for GBM treatment (Pal et al., 2018). Our research also revealed significant downregulation of CALCRL expression in ULMS; furthermore, CALCRL expression was positively correlated with CD4 memory resting T cells and resting mast cells while negatively correlating with CD8 T cells. As an immune-related gene, CALCRL holds promise for application in treating lymphedema and tumors.

As an inflammation-related gene, CALCRL collaborates with RAMP-1 to generate CGRP, which plays a crucial role in managing harmful and inflammatory stimuli during infections caused by uropathogenic *Escherichia coli* (UPEC) in the urinary tract (Montalbetti et al., 2023). The dynamic regulation of the adrenomedullary receptor, which was generated from the interaction between CALCRL and RAMP-2, has also been demonstrated to suppress inflammatory responses in septic shock (Dackor and Caron, 2007). Furthermore, CALCRL is considered to possess predictive potential for cancer susceptibility, treatment response, and prognosis. Huanhuan Hu et al. employed bioinformatics techniques to identify 11 inflammation-related genes, including CALCRL, for developing a breast cancer risk profile model aimed at predicting survival and prognosis (Hu et al., 2024). Reema B. Davis et al. reported that CALCRL deficiency exacerbates intestinal inflammation while inducing systemic lymphatic dysfunction and intestinal lymphangiectasis (Davis et al., 2017). Moreover, a recent study demonstrated that adrenomedullin 2, a CGRP-related neuropeptide, promotes tissue-protective ILC2 responses and limits intestinal inflammation (Uddin et al., 2025), further underscoring the role of CALCRL-mediated signaling in inflammatory regulation Our study similarly observed a significant reduction in CALCRL expression in ULMS; thus, inducing the signaling pathway mediated by CALCRL may facilitate inflammation resolution and enhance therapeutic efficacy against ULMS.

The molecular regulatory mechanisms, as well as the associated molecules and pathways of CALCRL in various diseases, are likely to be diverse. 1) GPCR-Mediated Signaling: Calcitonin gene-related peptide (CGRP), a short-lived osteopromotive neurotransmitter, is upregulated in the early stage of bone fracture, whereas the expression level of calcitonin receptor-like (CALCRL) is downregulated. These findings suggest that activation of the CALCRL-mediated signaling pathway may play a more critical role in facilitating tendon-bone healing (Zhao et al., 2024). 2) Potential Interaction with Tumor Microenvironment: Our immune infiltration analysis revealed that CALCRL expression is positively correlated with CD4^+^ memory resting T cells and resting mast cells, and negatively correlated with CD8^+^ T cells. This suggests that CALCRL may modulate the tumor immune microenvironment by influencing immune cell recruitment or activation, thereby indirectly affecting tumor cell behavior. 3) Involvement in Inflammation-Related Pathways: KEGG enrichment analysis of DIIRGs, including CALCRL, showed significant enrichment in pathways such as cytokine-cytokine receptor interaction, TNF signaling, and IL-17 signaling. These pathways are closely linked to tumor progression and immune modulation. Although direct regulation of these pathways by CALCRL has not been established in ULMS, studies in other cancers [e.g., glioblastoma (Pal et al., 2018), acute myeloid leukemia (Grandits and Wieser, 2021)] have shown that CALCRL can influence chemoresistance, stemness, and apoptosis through downstream effectors such as Bcl-2, XRCC5, and JAK1 (Tang et al., 2023). 4) Hypothesis Based on Co-Expression with RAMP Proteins: The functional specificity of CALCRL is highly dependent on RAMP co-expression (Hagner et al., 2002). In uterine tissues, CALCRL has been shown to interact with RAMP1 and RAMP2, forming receptors that respond to CGRP and adrenomedullin, respectively (Nikitenko et al., 2001). These ligands have been implicated in hypoxia adaptation, cell survival, and lymphatic regulation—processes relevant to ULMS biology. Recent evidence further demonstrates RAMP1-dependent hormonal regulation of CGRP signaling in the trigeminal ganglion (Holm et al., 2025), highlighting the tissue-specific modulation of CALCRL function through RAMP interactions.

Beyond its diagnostic utility, our findings suggest that CALCRL holds promise as a therapeutic target with broader clinical translational value. The significant correlation between CALCRL expression and immune cell populations—positive with CD4^+^ memory resting T cells and resting mast cells, negative with CD8^+^ T cells—suggests that CALCRL may shape the tumor immune microenvironment in ULMS, raising the possibility that targeting CALCRL could reverse immunosuppression and enhance anti-tumor immunity, potentially rendering ULMS more susceptible to immune checkpoint inhibitors (Rui et al., 2023; De Wispelaere et al., 2021). As a GPCR, CALCRL is highly druggable, with approximately 34% of FDA-approved drugs targeting this protein class (Hauser et al., 2017), offering multiple therapeutic avenues including agonist-based strategies to restore potential tumor-suppressive signaling [as demonstrated in glioblastoma (Pal et al., 2018)], antagonist-based approaches for contexts where CALCRL is upregulated [e.g., NSCLC (He and Ling, 2021)], AML [Gluexam et al., 2019; Grandits and Wieser, 2021)], and peptide-based therapeutics using its natural ligands CGRP and adrenomedullin, which are already in clinical development for other indications (Edvinsson et al., 2018). Furthermore, CALCRL-targeted therapy could synergize with other modalities such as immune checkpoint inhibitors, chemotherapy, or anti-inflammatory agents, the latter supported by evidence that combining GPCR modulation with COX-2 inhibition can enhance immunotherapy efficacy (Pelly et al., 2021). Beyond direct targeting, CALCRL expression could serve as a predictive biomarker for immunotherapy selection, prognostic stratification, or treatment monitoring via non-invasive imaging. However, clinical translation requires addressing challenges including the context-dependent biology of CALCRL (Wende et al., 2023), mechanistic validation through gain-/loss-of-function studies in ULMS models, development of specific modulators distinguishing between its RAMP-dependent functional complexes (Hagner et al., 2002; Nikitenko et al., 2001), and prospective biomarker validation in well-annotated cohorts. In summary, our findings position CALCRL not only as a diagnostic biomarker but also as a molecule with substantial therapeutic potential, providing a foundation for future investigations aimed at developing CALCRL-targeted therapies for ULMS.

Several limitations of this study should be acknowledged. First, regarding data sources and model robustness, our diagnostic model was developed using a single transcriptomic dataset (GSE64763) due to the rarity of ULMS. Although regularized machine learning algorithms (LASSO, mSVM-RFE) were employed to mitigate overfitting, and external validation was performed on a merged cohort comprising three independent datasets (GSE9511, GSE68295, GSE36610) with batch effect correction (SVA package), the possibility of overfitting and residual technical variability cannot be entirely excluded. Moreover, our model relies on a single gene (CALCRL), which—while rigorously validated—may be inherently sensitive to batch effects and technical variations across platforms. As demonstrated in other contexts, gene-pair ratio-based models offer superior stability through internal normalization (Xie et al., 2022). Future studies should develop more robust frameworks, such as gene-pair signatures, to enhance cross-platform reliability. Second, regarding clinical validation and translatability, the immunohistochemistry validation was performed on a single-center cohort (21 ULMS, 38 ULM) without stratification by clinicopathological features (e.g., stage, grade), limiting assessment of CALCRL’s correlation with disease aggressiveness. Additionally, the diagnostic cut-off values were optimized within each individual dataset, precluding immediate clinical adoption. A standardized, universally applicable threshold is essential for clinical translation and requires rigorous multi-center validation accounting for platform and population variability (Zhang et al., 2022). Third, regarding mechanistic understanding and causality, our study establishes a strong association between low CALCRL expression and ULMS but does not demonstrate causality. The bioinformatic and correlative nature of our analyses means we cannot determine whether CALCRL downregulation drives ULMS pathogenesis or is a consequence thereof. While KEGG enrichment implicated pathways such as IL-17 signaling and cytokine-cytokine receptor interaction, the direct regulatory relationship between CALCRL and these pathways remains unvalidated. Furthermore, the precise molecular mechanisms—whether through GPCR signaling, immune modulation, or other routes—are unverified due to the lack of functional experiments. Future investigations should employ causal inference approaches [e.g., Mendelian randomization (Zhang et al., 2024)] and direct gain-/loss-of-function studies in ULMS models to establish causality and mechanistic insights.

## Conclusion

Utilizing bioinformatics analysis techniques, we have validated the potential of CALCRL as a clinical diagnostic biomarker associated with immune and inflammatory responses in ULMS. This work offers valuable insights for the precise diagnosis and development of innovative therapeutic strategies for ULMS in the future.

## Data Availability

The datasets presented in this study can be found in online repositories. The names of the repository/repositories and accession number(s) can be found in the article/Supplementary Material.
