# Supplementary material for: Identification of the key immune and inflammatory related gene CALCRL as diagnostic biomarker in differentiating uterine leiomyosarcoma from leiomyoma
Source: Front Cell Dev Biol. 2026 Apr 10;14:1777897. doi: 10.3389/fcell.2026.1777897 (PMC13106448; doi:10.3389/fcell.2026.1777897)
Supplement: Supplementary file 1 [file DataSheet1.zip › supplementary material/Table S3 Detailed Parameters for Bioinformatics Analyses. .docx]

****Table: Detailed Parameters for Bioinformatics Analyses****

| ****Analysis Method**** | ****Software/Package**** | ****Key Parameter Settings**** | ****Purpose & Description**** |
| --- | --- | --- | --- |
| ****Differential Expression Analysis**** | R package Limma | • ****Filtering Threshold:**** \|log2 Fold Change (FC)\| > 0.585 • ****Significance Criterion:**** adj.P.Val < 0.05 | The limma package was used to compare ULMS and ULM samples in the GSE64763 dataset based on a generalized linear model and empirical Bayes method. \|log2FC\| > 0.585 corresponds to a 1.5-fold change in expression, aiming to screen for genes with biological significance. adj.P.Val was corrected for multiple hypothesis testing using the Benjamini-Hochberg method to control the false discovery rate. |
| ****LASSO Regression**** | R package glmnet | • ****Parameter alpha:**** alpha = 1 • ****Cross-validation:**** 10-fold cross-validation • ****Penalty parameter λ selection:**** lambda.min (the λ value that minimizes the mean cross-validated error) | LASSO (Least Absolute Shrinkage and Selection Operator) regression was used for feature selection to prevent model overfitting. alpha = 1 specifies LASSO regression. Through 10-fold cross-validation, the model's performance under different λ values was evaluated on the training data. The optimal λ value (lambda.min) that minimizes the model's deviance was ultimately selected to identify the feature genes most contributive to ULMS classification. |
| ****SVM-RFE Algorithm**** | R package e1071 (with custom functions from geoImmune16.msvmRFE.R) | • ****Algorithm:**** Multiple Support Vector Machine Recursive Feature Elimination (mSVM-RFE) as implemented in the source script • ****Cross-validation:**** 10-fold cross-validation (based on nfold=10 in the script) • ****Resampling details:**** The script uses folds to split data into 10 subsets for iterative feature elimination; however, repetition (e.g., 5 times) was not explicitly coded in the provided main script. • ****Kernel function:**** Not explicitly specified in the main script; likely defaults to radial basis function within the called custom functions. | The mSVM-RFE algorithm was used for stable feature selection. This method evaluates feature importance through recursive feature elimination combined with cross-validation. In the script, 10-fold cross-validation was applied to assess feature subsets and select the optimal gene set for distinguishing ULMS from ULM. The algorithm aims to reduce overfitting by leveraging resampling during feature selection. |
| ****CIBERSORT Immune Infiltration Analysis**** | R package CIBERSORT (with source script geoImmune26.CIBERSORT.R) | • ****Algorithm:**** Support Vector Regression • ****Reference Matrix:**** LM22 gene signature set (comprising signature genes for 22 immune cell subtypes) • ****Permutations:**** 1000 permutations | The CIBERSORT algorithm was used to quantify the relative abundances of 22 immune cell types in ULMS and ULM samples based on gene expression data. Support vector regression was used for the deconvolution calculation. The statistical significance (P-value) of the immune infiltration results for each sample was assessed through 1000 permutation tests to ensure the reliability of the findings. Results with P-value < 1 were retained (effectively all samples). |
